# Soil fertility relates to fungal-mediated decomposition and organic matter turnover in a temperate mountain forest

**DOI:** 10.1111/nph.17421

**Published:** 2021-05-20

**Authors:** Mathias Mayer, Boris Rewald, Bradley Matthews, Hans Sanden, Christoph Rosinger, Klaus Katzensteiner, Markus Gorfer, Harald Berger, Claudia Tallian, Torsten W. Berger, Douglas L. Godbold

**Affiliations:** 1 Forest Soils and Biogeochemistry, Swiss Federal Institute for Forest, Snow and Landscape Research (WSL), Zürcherstrasse 111, Birmensdorf 8903, Switzerland; 2 Department of Forest and Soil Sciences, Institute of Forest Ecology, University of Natural Resources and Life Sciences (BOKU), Peter-Jordan Straße 82, Vienna 1190, Austria; 3 Environment Agency Austria, Spittelauer Lände 5, Vienna 1090, Austria; 4 Terrestrial Ecology Group, Institute of Zoology, University of Cologne, Zülpicher Straße 47b, Cologne 50674, Germany; 5 Bioresources Unit, Center for Health & Bioresources, Austrian Institute of Technology GmbH (AIT), Konrad-Lorenz-Straße 24, Tulln 3430, Austria; 6 Symbiocyte, Vorgartenstraße 145, Vienna 1020, Austria

**Keywords:** carbon cycle, ectomycorrhizal fungi, Ellenberg indicator values, enzymes, fungal guilds, Gadgil effect, plant-soil feedback, priming

## Abstract

- Fungi are known to exert a significant influence over soil organic matter (SOM) turnover, however understanding of the effects of fungal community structure on SOM dynamics and its consequences for ecosystem fertility is fragmentary.
- Here we studied soil fungal guilds and SOM decomposition processes along a fertility gradient in a temperate mountain beech forest. High-throughput sequencing was used to investigate fungal communities. Carbon and nitrogen stocks, enzymatic activity and microbial respiration were measured.
- While ectomycorrhizal fungal abundance was not related to fertility, saprotrophic ascomycetes showed higher relative abundances under more fertile conditions. The activity of oxidising enzymes and respiration rates in mineral soil were related positively to fertility and saprotrophic fungi. In addition, organic layer carbon and nitrogen stocks were lower on the more fertile plots, although tree biomass and litter input were higher. Together, the results indicated a faster SOM turnover at the fertile end of the gradient.
- We suggest that there is a positive feedback mechanism between SOM turnover and fertility that is mediated by soil fungi to a significant extent. By underlining the importance of fungi for soil fertility and plant growth, these findings furthermore emphasise the dependency of carbon cycling on fungal communities below ground.

## Introduction

The fertility of forest soils is a fundamental factor governing plant growth and ecosystem carbon (C) balances (Vicca *et al.*, 2012; Binkley & Fisher, 2013). Fertility is an ecosystem characteristic describing the relative availability of below-ground resources required for plant growth (Chapin III *et al.*, 2002), and therefore depends on soil properties such as water availability and the supply of mineral nutrients mobilised from both weathering of the geological substrate and turnover of soil organic matter (SOM) (Hansson *et al.*, 2020). Fertile soil conditions have been associated with higher SOM decomposition rates and therefore a faster recycling of organic nutrients back to the plants (Sariyildiz & Anderson, 2003; Kyaschenko *et al.*, 2017b, 2019). A positive feedback between fertility and SOM turnover may explain why more fertile and productive forests were shown to feature a lower soil C accumulation than less fertile and less productive forests (Vesterdal & Raulund-Rasmussen, 1998; Ladegaard-Pedersen *et al.*, 2005; Hansson *et al.*, 2020). Recently, fungal-mediated decomposition and interactions among fungal guilds were identified to link soil fertility and SOM turnover in a boreal forest (Kyaschenko *et al.*, 2017b). Whether this holds true for other forest ecosystems is, however, unknown.

In soils of temperate and boreal forests, saprotrophic and ecto-mycorrhizal fungi play a pivotal role in the transformation and decomposition of SOM (Fernandez & Kennedy, 2016; Crowther *et al.*, 2019; Frey, 2019; Zak *et al.*, 2019). Both fungal guilds can acquire SOM-bound resources (i.e. C and nutrients) by releasing hydrolytic and oxidative enzymes that break down organic compounds (Baldrian, 2008; Schneider *et al.*, 2012; Kohler *et al.*, 2015). In contrast with saprotrophic fungi, ectomycorrhizal fungi largely lack the genetic capacity to fully metabolise soil C (Baldrian, 2009; Lindahl & Tunlid, 2015; Frey, 2019; Zak *et al.*, 2019). Instead, ectomycorrhizal fungi form symbioses with plants and receive photosynthetically assimilated C from their host in exchange for soil-derived nutrients (Read & Perez-Moreno, 2003; Van Der Heijden *et al.*, 2015).

Suppression of saprotrophic activity by ectomycorrhizal fungi through competition for limiting nutrients (particularly nitrogen, N) has been proposed to lead to lower SOM decomposition, a mechanism referred to as the ‘Gadgil effect’ (Gadgil & Gadgil, 1971; Fernandez & Kennedy, 2016). The suppression of saprotrophic decomposition by ectomycorrhizal fungi was thereby suggested to relate to lower soil CO_2_ losses, increased soil C accumulation and wider soil C : N ratios (Orwin *et al.*, 2011; Averill & Hawkes, 2016; Sterkenburg *et al.*, 2018; Smith & Wan, 2019). Ectomycorrhizal constraints on saprotrophic decomposition were demonstrated to be most pronounced in the litter layer and in upper soil horizons with abundant amounts of energy-rich substrate (Brzostek *et al.*, 2015; Averill & Hawkes, 2016; Fernandez & Kennedy, 2016; Sterkenburg *et al.*, 2018). Moreover, the occurrence of a Gadgil effect seems to be strongly determined by substrate quality; recent studies have suggested that ectomycorrhizal suppression of decomposition prevails in low quality substrates with high C : N or lignin : N ratios, respectively (Smith & Wan, 2019; Fernandez *et al.*, 2020). These findings comply with an earlier hypothesis by Fernandez & Kennedy (2016) that ectomycorrhizal constraints on saprotrophs are more likely when soil fertility and associated N availability is low.

As a mechanism to acquire nutrients, ectomycorrhizal fungi and their host roots have also been proposed to be actively involved in SOM decomposition through the release of easily degradable carbohydrates into the rhizosphere, a process known as ‘priming’ (Phillips *et al.*, 2012; Frey, 2019; Zak *et al.*, 2019). Priming was suggested to stimulate saprotrophic decomposition in the litter layer as well as stronger degraded organic or mineral soil layers (Fontaine *et al.*, 2007; Brzostek *et al.*, 2015; Kohout *et al.*, 2018; Fernandez *et al.*, 2020). This mechanism is thought to increase soil CO_2_ losses - by an estimated 12% in temperate forests (Bastida *et al.*, 2019) - thereby reducing soil C stores (Huo *et al.*, 2017; Guenet *et al.*, 2018). The process of priming has been shown to increase with above-ground plant biomass and increased tree productivity (Hoosbeek *et al.*, 2004; Huo *et al.*, 2017).

Previous studies conducted in forests have found support for both the Gadgil effect and priming, primarily by building on results derived from manipulation experiments that reduced C flow to roots, such as root trenching, tree girdling, or clear cutting (Brzostek *et al.*, 2015; Averill & Hawkes, 2016; Kohout *et al.*, 2018; Sterkenburg *et al.*, 2018; Fernandez *et al.*, 2020). Yet, the proposed mechanisms of fungal-mediated decomposition and interguild fungal interactions have rarely been used to explore soil fertility-SOM feedback (Sterkenburg *et al.*, 2015; Kyaschenko *et al.*, 2017b). Our mechanistic understanding of how fungi affect SOM dynamics at an ecosystem level is therefore still limited (Fernandez & Kennedy, 2016; Frey, 2019; Zak *et al.*, 2019).

**Fig. 1:**
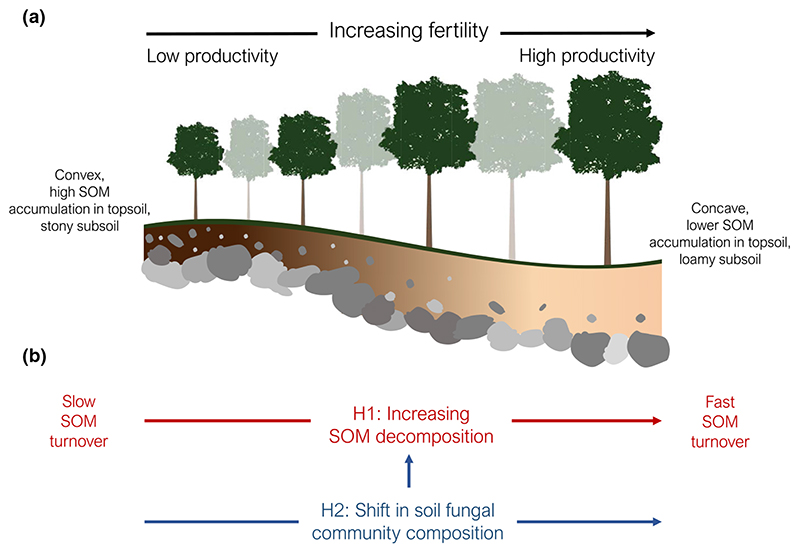
(a) Conceptual outline and characterisation of the studied fertility gradient in a beech dominated mountain forest and (b) the hypotheses predicting (H1) increasing soil organic matter (SOM) decomposition with increasing fertility, and (H2) fertility coincides with a shift in the soil fungal community composition that consequently influences SOM decomposition dynamics.

Here, we studied a natural fertility gradient in a mountain forest of European beech that is typical for the temperate montane region of Central Europe. Less fertile plots at the site are characterised by a low stand productivity, topsoils with a high organic matter accumulation, and stony subsoils, while more fertile plots featured a higher productivity, topsoils with lower organic matter accumulation and loamy subsoils (Fig. 1a). The general aim of this study was to evaluate the interrelation between fertility, SOM turnover and soil fungi in forest decomposition dynamics. Based on earlier findings (Ladegaard-Pedersen *et al.*, 2005; Sterkenburg *et al.*, 2015; Kyaschenko *et al.*, 2017b, 2019) we hypothesised that: (1) SOM decomposition is positively related to fertility, resulting in faster SOM turnover under fertile conditions with higher litter input; and (2) fertility coincides with a shift in the soil fungal community composition that consequently influences SOM decomposition dynamics (Fig. 1b). Specifically, we discuss whether fungal interguild competition for limiting nutrients (i.e. a Gadgil effect) and/or stimulation of saprotrophic activity (i.e. priming) may be potential mechanisms underlying SOM decomposition dynamics along the fertility gradient.

The fertility gradient was quantified by analysing stand, organic layer and mineral soil properties (e.g. litter input, C and N stocks). Soil was further analysed for potential enzymatic activities, CO2 losses from microbial respiration, fungal community composition and interrelations between these variables. Enzymatic activities, microbial respiration and mean residence time of the organic layer were used as proxies for SOM decomposition. The assessment of fertility was based on vascular plant species and associated Ellenberg indicator values (Ellenberg *et al.*, 1992).

## Materials and Methods

### Site description

The study was conducted in the Reichraminger Hintergebirge, a mountain range located in the Austrian Calcareous Alps (47°49’08”N, 14°23’34”E). The forest stand is dominated by European beech (*Fagus sylvatica* L.) with a stand age of *c*. 146 yr in 2015. The site is southeast exposed with a slope inclination of 35° at an elevation of 1000 to 1100 m above sea level (asl) (Supporting Information Fig. S1). The parent bedrock is limestone, the dominant soil types are Rendzic Leptosol and Chromic Cam-bisol. Average annual air temperature and precipitation are 7.8°C and 1645 mm, respectively (Kobler *et al.*, 2015). Sixteen plots (c. 25 m × 25 m) were selected along a natural fertility gradient (extent *c*. 700 m; Fig. S1), characterised by a change in topography, soil type and depth, and hydrology. The fertility gradient followed the contour line, with less fertile plots featuring a convex topography, shallower, stony soils and more fertile plots holding deeper, loamy soils and a concave topography (Fig. 1a).

### Stand inventory, vegetation survey, microclimatic measurements and litter fall collection

In May 2015, diameter at breast height and height of trees were measured at each plot; allometric functions were applied to calculate above-ground woody biomass of trees (Wutzler *et al.*, 2008). For each plot, the identity and dominance of plant species in the herbal layer was determined at four subplots; species dominance was visually estimated and expressed in percentage soil cover. Subplots were defined as triangular areas between three beech trees each. Plant species were assigned Ellenberg indicator values (Table S1) and cover-weighted mean indicator values were calculated per plot (Ellenberg *et al.*, 1992). Measurements of soil temperature (5 cm depth) and moisture (0-7 cm depth) were conducted six times between May and August 2015 using a handheld thermometer and a TDR moisture meter (Spectrum Technologies, Aurora, IL, USA); plot-wise means of the six measurement campaigns were used for further analyses. In total, 20 litter traps (d: 52 cm, *h:* 70 cm) were installed along the gradient in September 2015. Traps were installed in close vicinity to the established plots (Fig. S1). Until October 2016, litter traps were emptied regularly and collected litter was dried (105°C) and weighed (± 0.01 g); litter fall was subsequently summed up for each trap and is given in gm^—2^yr^—1^. Depending on the location along the gradient, litter traps were grouped into three levels of fertility for further analyses (low, medium, high fertility; Fig. S1).

### Organic layer and mineral soil sampling and processing

In June 2015, organic layer samples were taken at 12 selected plots along the gradient (Fig. S1, dotted line rectangles). Litter (OLF) and humus horizons (OH, if present) were sampled separately with a large corer (*d*: 19 cm). Four replicates were taken per plot. Samples were pooled per plot and horizon, and roots and stones were removed. Total mass of litter and humus samples was determined by weighing (± 0.01 g) and subsequent multiplication with weight-conversion factors determined for subsamples (105°C, 48 h).

In August 2015, mineral soil samples were taken at each plot along the gradient (Fig. S1, solid line rectangles). Samples were taken from the mineral topsoil (0-10 cm, *c*. 1 l soil volume) using a shovel. Four replicates were taken per plot. Due to the high stone content (up to 80% of soil volume), sampling deeper than *c*. 10 cm was hardly feasible at many locations. Topsoil was, however, the largest pool for organic C and N (see Methods S1;Table S2). Soil samples were immediately sieved (2 mm) in the field. For fungal community analysis, 0.5 g of homogenised mineral soil was weighed into 1.5 ml LifeGuard Soil Preservation Solution (MO BIO, Carlsbad, CA, USA). Mineral soil for enzyme analyses was frozen at —20°C on the same day, and soil for other analyses was kept at 4°C until further processing.

To determine mineral soil bulk density, stone content and root biomass, three small soil pits (c. 15 cm × 15 cm surface area) were dug down to 10 cm mineral soil depth per plot. After the organic layer was removed, the mineral soil was sampled; the sampled soil volume was estimated by refilling the pit with quartz sand and measuring the volume sand used. In the laboratory, samples were weighed; roots and stones were picked per hand and rinsed clean. Fine roots (*d*< 2 mm), coarse roots (*d*> 2 mm), stones, and subsamples (*c*. 10 g) of sieved (2 mm) soil were dried (105°C, 24 h) and weighed (± 0.0001 g). Root biomass volume (g m^—2^) and stone content (vol%) were determined using dry weight and specific densities. Fine soil dry mass (gm^—2^) and fine soil bulk density (g cm^—3^) was subsequently calculated for each plot. Coarse roots were not used for statistical analysis.

### Organic layer and mineral soil analyses

Total C and N concentration (%) of organic layer and mineral soil samples was analysed on a 300 mg subsample using a TruSpec CHN analyser (Leco, St Joseph, MI, USA); subsamples were dried (105°C, 24 h) and ground before analysis (Pulverisette 5; Fritsch, Germany). Carbon and N stocks of the organic layer were subsequently calculated by multiplying the total dry mass of the horizon by C and N concentrations. Inorganic C concentration of mineral soil subsamples was determined using the Scheibler method (ONORM L 1084, 1999). Organic soil C concentration of mineral soil layers was calculated as the difference in inorganic and total C concentrations. Mineral soil organic C and N stocks (0-10 cm; gm ^2^) were calculated from respective organic C and N concentrations multiplied by dry weight of mineral soil.

The mean residence time of the organic layer was calculated per plot (Berger *et al.*, 2009). For that, the organic layer dry mass was divided by the average annual litter fall rate of the closest litter traps (Fig. S1). This approach assumes root litter input into the organic layer to be negligible.

Soil CO_2_ efflux from microbial respiration was measured on fresh mineral soil (equivalent to *c*. 25 g oven-dried soil) within a few days after sampling. For respiration measurements, soil was sieved to 2 mm and filled in 200 cm^3^ steel cylinders at field bulk density (Reichstein *et al.*, 2000; Schindlbacher *et al.*, 2015). After c. 3 d of equilibration at 4°C, cylinders were placed into 2 l plastic containers connected to an infrared gas analyser unit (SBA-4; PP Systems International, Amesbury, MA, USA). In brief, microbial respiration of each sample was determined as ΔCO_2_ within closed containers for Δ6 min. Microbial respiration was determined at a standardised temperature of 15°C. Respiration rates are expressed in mg C g ^-1^ C d and in mg Cm ^-2^d ^-1^ using total dry mass of mineral soil for conversion. Details on the measurement system and protocol can be found elsewhere (Mayer *et al.*, 2017a,b).

Potential activities of hydrolytic soil enzymes were measured fluorometrically according to Marx *et al.* (2001) and German *et al.* (2011). Briefly, 0.5 g of mineral soil was suspended in 50 ml of a 100 mM Tris buffer, pH 7.5, and homogenised for 1 min in a sonication bath (48 kHz, 50 W). Aliquots of 200 μl were pipetted under constant stirring (on a magnetic plate) into black 96- well microplates, with four technical replicates for each sample. Optimal substrate concentrations and incubation times for leucine aminopeptidase (1 mM), *N*-acetyl-β-D-glucosaminidase (1 mM), β-glucosidase (0.5 mM), acid phosphatase (2mM), β- xylosidase (1 mM) and cellobiohydrolase (0.3 mM) were evaluated in advance to avoid potential substrate inhibition. Next, 50 μl of substrate (dissolved in deionised water) were added to each well and the plate shaken horizontally for 30 s for mixing. The microplates were incubated at 20°C in the dark for 120 min (acid phosphatase) or 180 min (for all other enzymes). Fluorescence was measured using a multiplate reader with an excitation of 365 nm and an emission of 450 nm, at 20 and 100 flashes (EnSpire; Perkin Elmer, Waltham, MA, USA). Standard curves were prepared in buffer solution using four standard solutions with concentrations between 10 μM and 250 μM, for substrates based on 4-methylumbelliferone (all enzymes except leucine aminopeptidase) and two standard curves with concentrations of 20 μM and 50 μM, for substrates based on 7-amino-4- methylcoumarin (leucine aminopeptidase). Corresponding sets of standard curves were prepared in soil slurry to account for quenching. To measure potential phenol oxidase activity, 3,4- dihydroxy-L-phenylalanine (DOPA) was used as substrate. Here, 900 μl of soil suspension (or 900 μl of buffer solution for blank wells) were mixed with an equivalent amount of a 10 mM DOPA solution (prepared in 100 mM TRIS buffer), shaken horizontally for 10 min and centrifuged at c. 1500 ***g*** force for 5 min. Then, 250 μl of this suspension was transferred into a clear 96-well plate with three-fold repetition. Absorption was measured immediately (time 0), and after c. 6 h of incubation in the dark (20°C), at 450 nm using a multiplate reader (as above). Potential phenol oxidase activity was calculated as the difference between absorption at time 0 and after incubation. Potential activities of hydrolytic and oxidative enzymes are expressed in mmol or mol g^-1^ C h^-1^ and in mmol or mol m^-2^ h^-1^ using total dry mass of mineral soil for conversion.

### Soil fungal community analysis

For DNA isolation from mineral soil samples, half of the suspension in LifeGuard Soil Preservation Solution (see above) was transferred to the wells of a Bead Plate from the PowerSoil-htp 96 Well Soil DNA Isolation Kit (Mo Bio, Carlsbad, CA, USA). After centrifugation and removal of the supernatant, the combined vacuum and centrifugation protocol of the manufacturer was followed. Cell lysis was carried out in a FastPrep-96 bead beater (MP Biomedicals, Santa Ana, CA, USA) twice at 6 ms ^-1^ for 45 s with a 1 min break before the second lysis. To increase recovery of DNA from soil (Feinstein *et al.*, 2009), new Bead Solution and Solution C1 were added to the soil pellet after the first extraction and the full extraction was repeated. Library preparation and Illumina MiSeq sequencing of fungal amplicons was conducted as described in Keiblinger *et al.* (2018). In brief, the fungal ITS2 region was amplified with the primer pair of ITS3Mix_NeXTf and ITS4Mix_NeXTr. Forward and reverse primers were equimolar mixes of modified versions of original primers published by White *et al.* (1990) as suggested by Teder-soo *et al.* (2015). Nextera XT adapters were attached to the 5^0^- end of the fungal-specific primers for subsequent indexing and high-throughput sequencing. Illumina MiSeq PE250 sequencing was performed at the NGS Unit of the Vienna Biocenter Core Facility GmbH (Vienna, Austria). Quantification of total fungal DNA was carried out using qPCR with FungiQuant primers targeting the SSU region (Liu *et al.*, 2012) and following the protocol described in Unterwurzacher *et al.* (2018) with a modified assay volume of 10 μl. The qPCR standard was prepared by mixing equal amounts of genomic DNAs from *Penicillium canescens* NG_p02, *Trichoderma harzianum* NG_p29, and *Tritirachium* sp. gab0401. Total fungal DNA is expressed in μg DNA m ^-2^ using total dry mass of mineral soil for conversion. Primers for sequencing and qPCR targeted different regions (ITS2 and SSU, respectively) in the rRNA gene cluster due to different requirements for specificity and sequence variability. Both regions are present in the same copy number per genome.

Sequence data analysis followed the steps outlined in Unter-wurzacher *et al.* (2018) and Gorfer *et al.* (2021). USEARCH scripts were used for chimaera detection and filtering of underrepresented sequences (< 10 reads in the full dataset). VSEARCH (Rognes *et al.*, 2016) was used for clustering and counting sequences per cluster given a 97% sequence similarity, which compensated for an artificial inflation of operational taxonomic units (OTU) numbers. Taxonomic affiliation of OTUs was carried out with the UTAX script against the UNITE database (KÕljalg *et al.*, 2013), while manual editing of the data increased phylogenetic accuracy (Hofstetter *et al*., 2019). When no accurate classification at the genus level was possible, the closest taxonomic level, to which a clear affiliation was possible, was used instead. Nonfungal sequences were excluded from further analyses. Fungal OTUs were affiliated to ecological lifestyles/guilds (Deltedesco *et al*., 2020; Gorfer *et al*., 2021); the lifestyles/guilds are: ectomycorrhizal fungi, other symbiotic fungi (e.g. species with unspecific mycorrhizal lifestyle or forming arbuscular mycorrhizas), saprotrophic ascomycetes, saprotrophic basidiomycetes, other saprotrophic fungi (e.g. *Mortierella*, Rhizophydiales, *Mucor*), pathogenic fungi, and those of unknown lifestyle (Table S3). For statistical community analysis (see below), fungal OTUs were taxonomically grouped at genus level or closest higher taxonomic level (e.g. family). Ratios between relative abundances of ectomycorrhizal fungi and saprotrophic fungal guilds were calculated for each plot.

### Statistical analysis

All variables were averaged per plot before analyses. To assess soil fertility continuously, vascular plant’s Ellenberg indicator values for nutrients, soil reaction (a proxy for soil pH), and moisture were used. In brief, the indicator variables were analysed by means of principal component analysis (PCA; Fig. S2). The scores of the first PCA axis, integrating the availability of soil resources to plants, were used to represent a ‘fertility index’. See Methods S2 for a detailed description of the calculated fertility index.

Variables were related to each other using linear regression models. Models were extended by a variogram correlation structure when residuals were spatially autocorrelated (Zuur *et al.*, 2009). Differences in annual litter fall among fertility levels (Fig. S1) was tested by means of analysis of variance (ANOVA); to meet the criteria of ANOVA, data were log transformed before analysis.

Detrended correspondence analysis (DCA) was used to determine patterns among the fungal community in mineral soil (Paliy & Shankar, 2016). To study the effect of fertility on the fungal community, the fertility index was correlated to the DCA scores. To explore the role of fungi in SOM decomposition in greater detail, potential soil enzyme activities, microbial respiration, and mineral soil C : N ratios were additionally correlated to DCA scores. Canonical correspondence analysis (CCA) was used to investigate how much of the total variation was explained by the individual variables. The significances of the variables were tested by means of Monte Carlo permutation tests (n = 999). DCA and CCA were based on relative abundance of 352 taxonomic groups that occurred on < 3 plots.

Level of significance was set at *P*< 0.05. Statistical analysis and plotting was conducted in R (R Core Team, 2017) using packages NLME (Pinheiro *et al.*, 2014) and VEGAN (Oksanen *et al.*, 2016).

## Results

### Characteristics of the fertility gradient

**Fig. 2:**
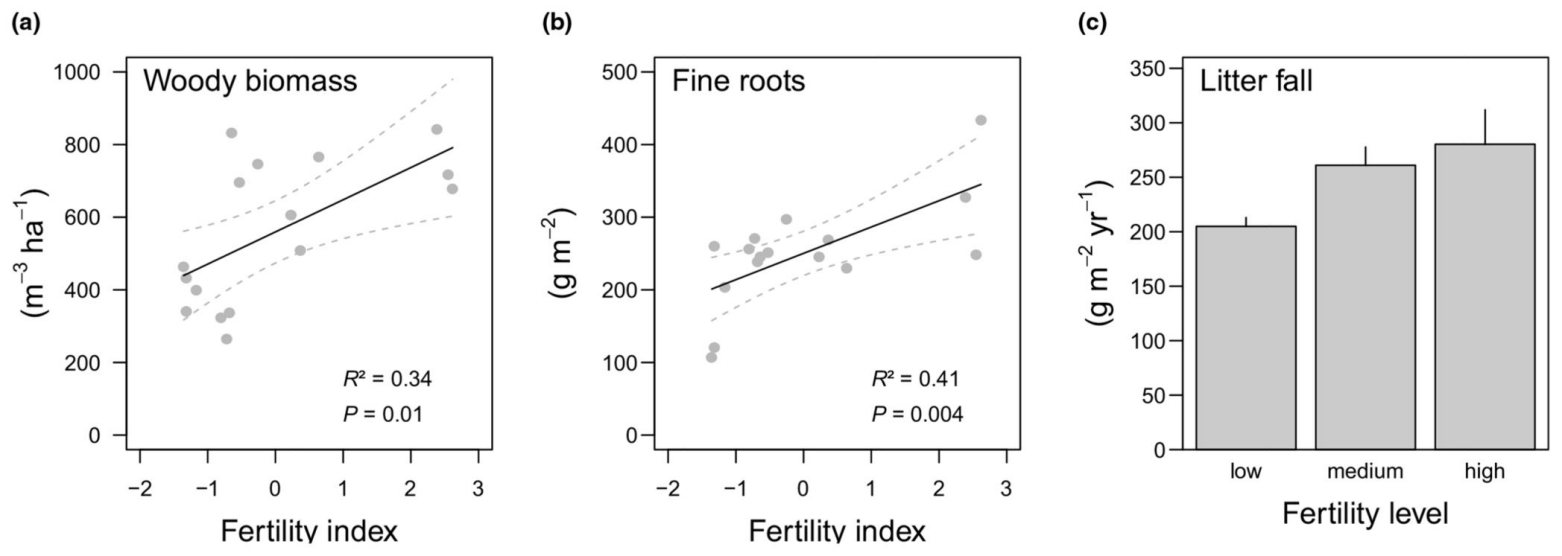
Relationship between the fertility index and fertility levels of a beech dominated mountain forest and (a) above-ground woody biomass of trees, (b) fine root biomass, and (c) annual leaf litter fall, respectively. The fertility index is based on the first axis of a principal component analysis of Ellenberg indicator values for vascular plants at sampling plots (Supporting Information Fig. S2). Given are test statistics of linear regression models (n = 16). Solid and dashed lines show fitted models and 95% confidence intervals, respectively.

The investigated fertility gradient was characterised by plant species such as *Carex alba*, *Helleborus niger*, and *Daphne laureola* at less fertile plots, and *Mercurialis perennis* and *Allium ursinum* at more fertile plots (Table S1). A particularly high abundance of *Allium ursinum* indicates a high nutrient and moisture availability. The fertility index (from this point forwards referred to as ‘fertility’) was strongly related to vascular plant’s Ellenberg indicator values for soil nutrients and moisture (Fig. S2). Aboveground woody biomass of trees and fine root biomass were positively correlated with fertility (Fig. 2a,b), being *c*. 2-fold and 1.5- fold higher at the more fertile plots of the gradient when compared with the less fertile plots, respectively. Similarly, litter fall increased with increasing fertility levels (Fig. 2c; *F*_2,17_ = 3.687, *P* = 0.047).

**Fig. 3:**
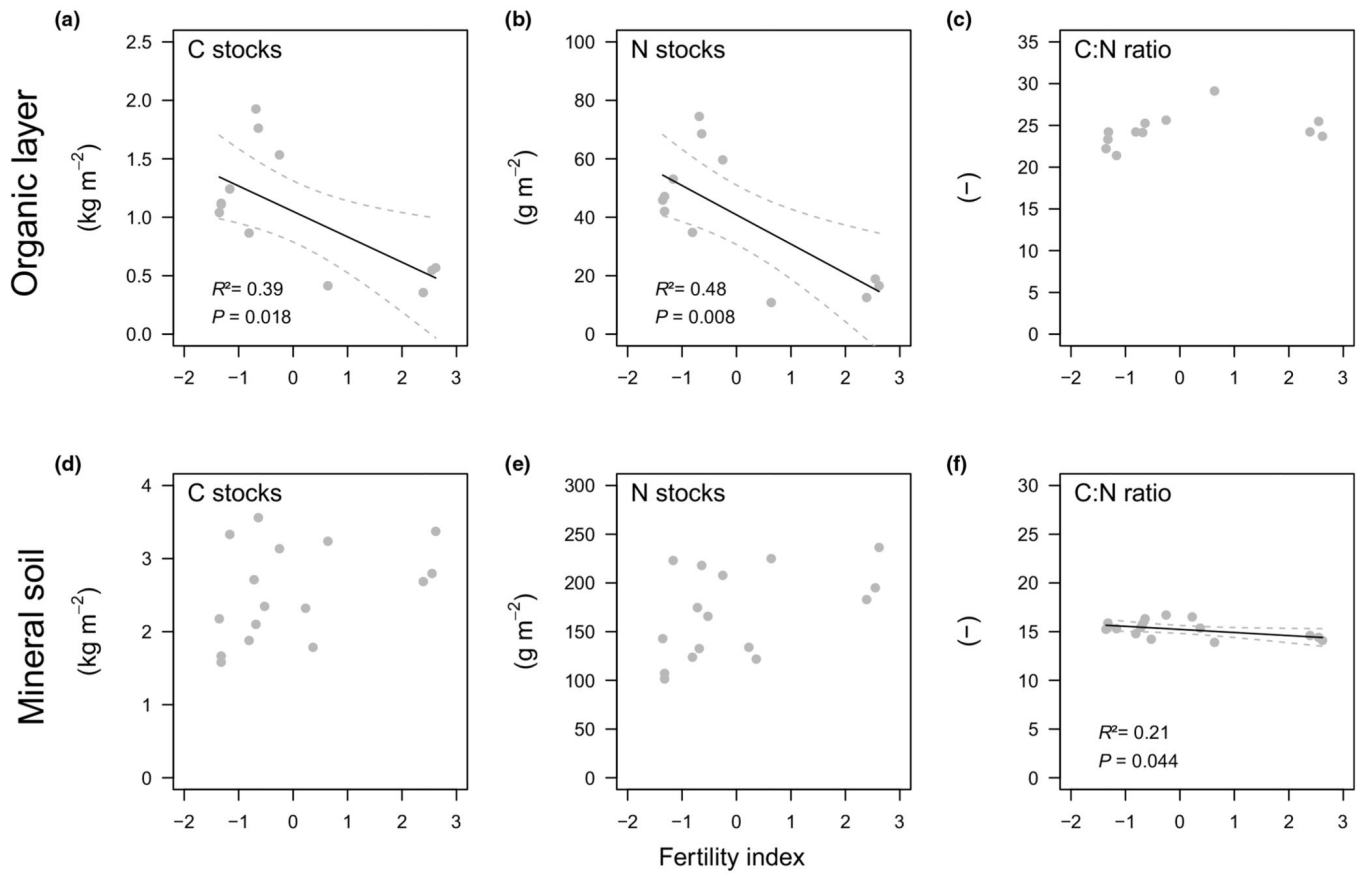
between the fertility index of a beech dominated mountain forest and carbon (C) and nitrogen (N) stocks, and C : N ratios of the organic layer (a-c) and the mineral soil (0-10cm) (d-f), respectively. The fertility index is based on the first axis of a principal component analysis of Ellenberg indicator values for vascular plants at sampling plots (Supporting Information Fig. S2). Given are test statistics of linear regression models (*n* = 16). Solid and dashed lines show fitted models and 95% confidence intervals, respectively.

Fertility was negatively correlated with organic layer C and N stocks (Fig. 3a,b). No effect of fertility was observed on litter layer C : N ratios (Fig. 3c). Due to the absence OH horizon, at some plots C : N ratios were not determined. Fertility was negatively correlated with mineral soil C and N concentrations (Fig. S3). Mineral soil C and N stocks were similar across the gradient (Fig. 3d,e). Fertility was negatively correlated with both mineral soil C : N ratios (Fig. 3f) and soil pH (Fig. S3); C : N ratios ranged between *c*. 15 and 16 at more fertile and less fertile plots, respectively.

Soil volumetric water content at 0-7 cm mineral soil depth was similar across the fertility gradient, and soil temperature at 5 cm depth was negatively correlated with fertility (Fig. S3).

### Fertility effects on decomposition processes and fungal guilds

**Fig. 4:**
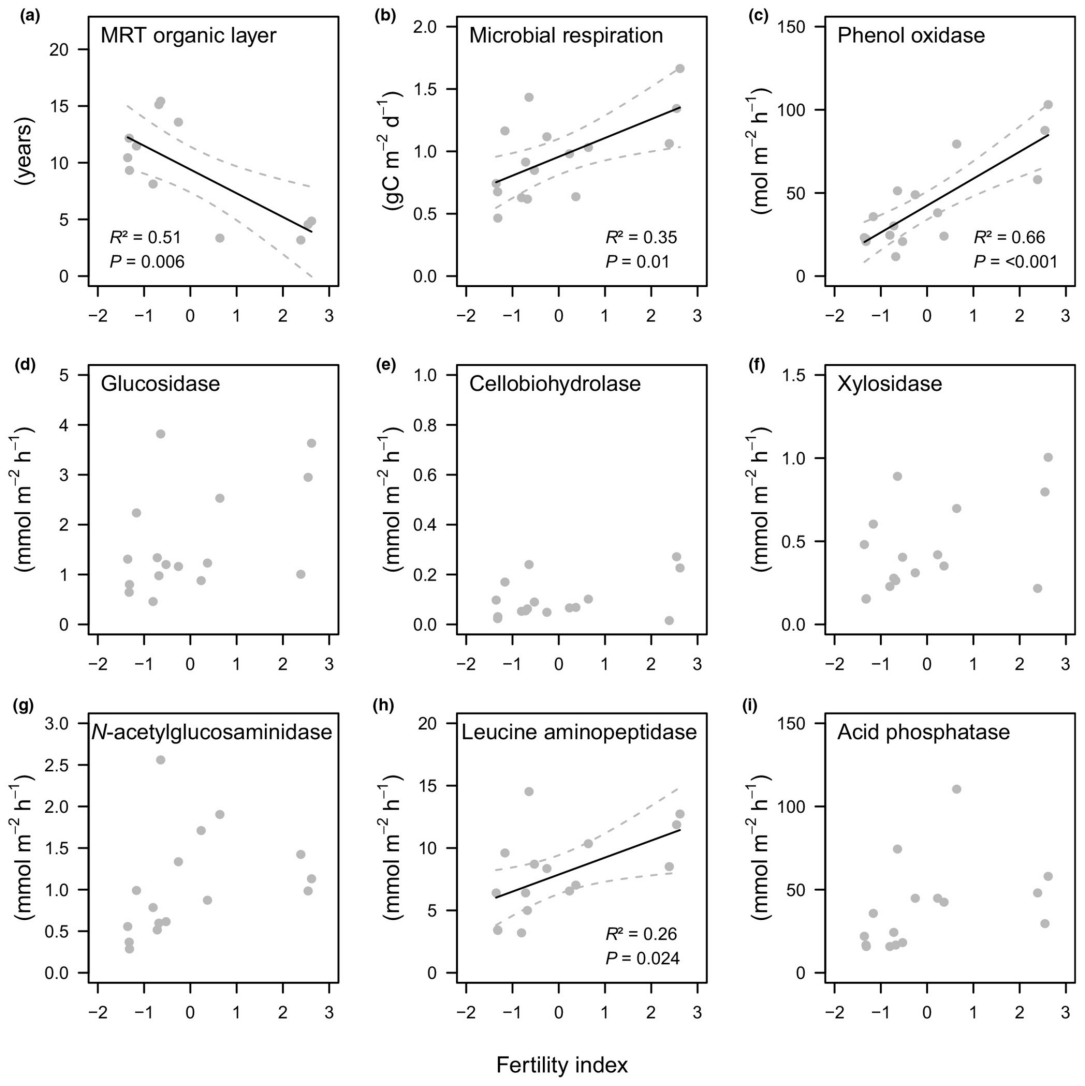
Relationship between the fertility index of a beech dominated mountain forest and (a) mean residence time (MRT) of the organic layer, (b) microbial respiration and the potential activity of (c) phenol oxidase, (d) ß-glucosidase, (e) cellobiohydrolase, (f) ß-xylosidase, (g) N-acetyl-glucosaminidase, (h) leucine aminopeptidase, and (i) acid phosphatase in the mineral soil (0-10 cm). The fertility index is based on the first axis of a principal component analysis of Ellenberg indicator values for vascular plants at sampling plots (Supporting Information Fig. S2). Given are test statistics of linear regression models (n = 16). Solid and dashed lines show fitted models and 95% confidence intervals, respectively.

Fertility was correlated with our proxies for SOM decomposition in both the organic layer, and the mineral soil. The mean residence time of the organic layer was negatively correlated with fertility and varied from *c*. 13 yr at less fertile plots to *c*. 5 yr at more fertile plots, respectively (Fig. 4a). A fertility effect on SOM decomposition in the mineral soil was identified by a positive correlation with the microbial respiration rates and the potential activities of phenol oxidase and leucine aminopeptidase (Fig. 4b,c,h). No correlation was found between fertility and the five other extracellular soil enzymes (Fig. 4).

Fertility effects on proxies for SOM decomposition in mineral soil were also given indirectly by correlations with above-ground woody biomass of trees. Above-ground woody biomass of trees was positively correlated with microbial respiration and the potential activities of all tested extracellular enzymes except cel- lobiohydrolase (Table 1).

**Fig. 5:**
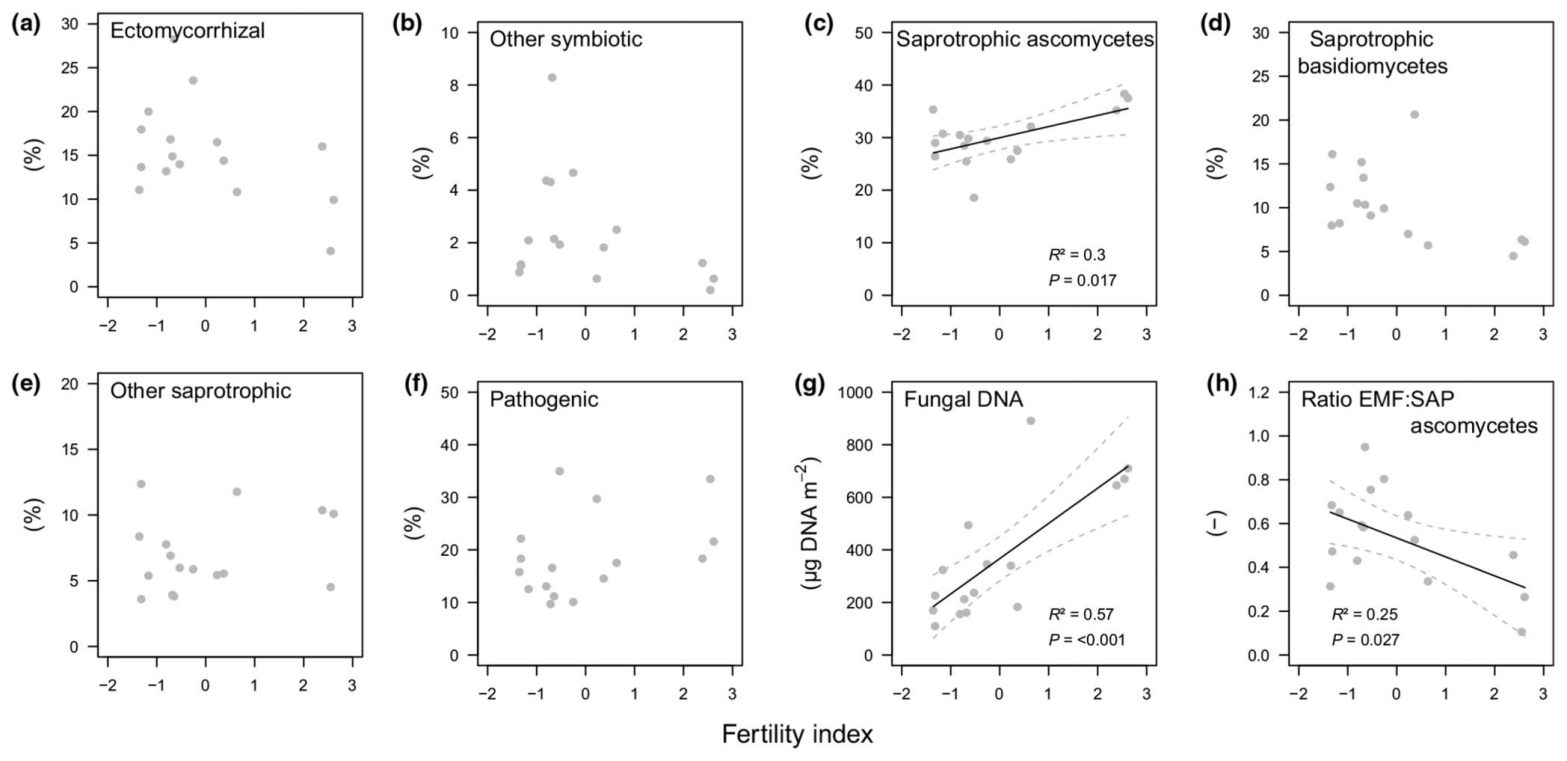
Relationship between fertility index of a beech dominated mountain forest and the relative abundance of (a) ectomycorrhizal fungi, (b) other symbiotic fungi, (c) saprotrophic ascomycetes, (d) saprotrophic basidiomycetes, (e) other saprotrophic fungi (e.g. moulds), (f) pathogenic fungi, (g) the total amount of fungal DNA, and (h) the ratio between relative abundance of ectomycorrhizal fungi (EMF) to saprotrophic (SAP) ascomycetes in the mineral soil (0—10 cm). The fertility index is based on the first axis of a principal component analysis of Ellenberg indicator values for vascular plants at sampling plots (Supporting Information Fig. S2). Given are test statistics of linear regression models (n = 16). Solid and dashed lines show fitted models and 95% confidence intervals, respectively.

Fertility was positively correlated with the relative abundance of saprotrophic ascomycetes while other fungal guilds were unaffected by fertility (Fig. 5). Fertility was positively correlated with total fungal DNA (Fig. 5g) and negatively correlated with the ratio of relative abundances of ectomycorrhizal fungi to sapro- trophic ascomycetes (Fig. 5h). Ratios between ectomycorrhizal fungi and other saprotrophic groups were not related to fertility (Fig. S4).

### Relationship between soil fungi, microbial respiration and enzyme activities

The relative abundance of saprotrophic ascomycetes was positively related to microbial respiration rates and phenol oxidase activity (Tables 2,S5). Saprotrophic basidiomycetes were negatively related to microbial respiration and phenol oxidase activity (Tables S4,S5). Other fungal guilds were neither related to microbial respiration nor to enzyme activities on an area-based unit (Table S4), however a negative correlation between these variables and the guild of other symbiotic fungi was found when units were expressed per g soil C (Table S5). Total fungal DNA was positively related to microbial respiration and all measured enzymes (Table 2).

### Fungal community composition

**Fig. 6:**
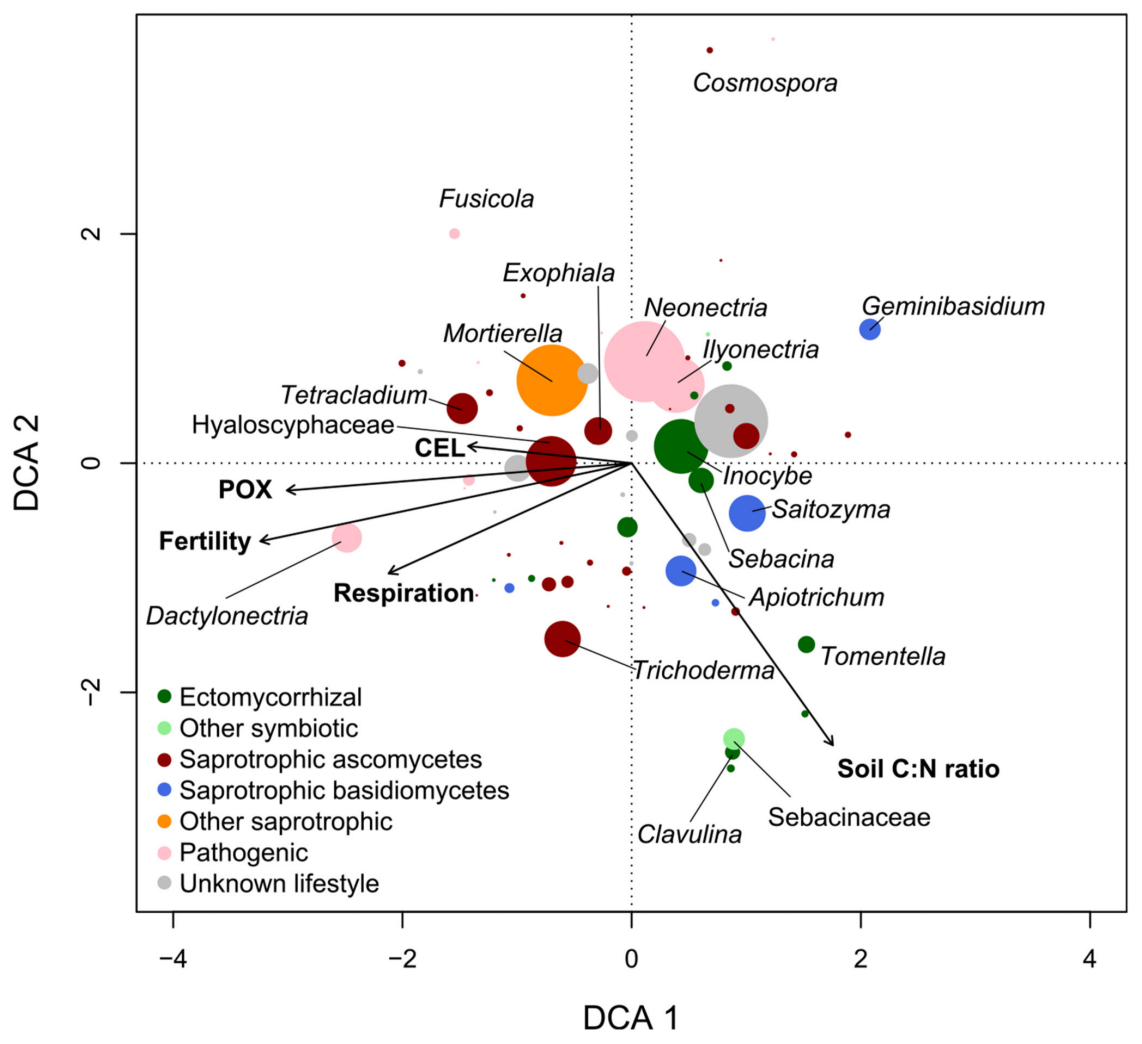
Patterns of variation in soil fungal community in the mineral soil (0-10 cm) across a fertility gradient in a beech dominated mountain forest as explained by detrended correspondence analysis (DCA). The DCA is based on 16 pooled sampling plots and 352 fungal taxonomic groups present at ≥ 3 sampling plots. Taxonomic grouping occurred at genus level or closest taxonomic level. Colour-coded fungal lifestyles/guilds were assigned; symbol size gives an indication for relative abundance. To increase readability, not all groups are labelled. Vectors are significant mineral soil variables related to fungal community patterns (POX, phenol oxidase; LEU, leucine aminopeptidase). Fertility is an index based on the first axis of a principal component analysis of Ellenberg’s indicator values for vascular plants at sampling plots (Supporting Information Fig. S2). Details on fungal taxonomic groups, relative abundances and associated lifestyles/guilds are given in Table S3.

The fungal community in the mineral soil was dominated by saprotrophic ascomycetes from the taxonomic groups of Hyaloscyphaceae, *Tetracladium* and *Trichoderma*, pathogenic fungi from *Neonectria*, *Dactylonectria and Ilyonectria*, ectomycorrhizal fungi from *Inocybe*, and *Sebacina* and other symbiotic fungi from the unspecific mycorrhizal group of Sebacinaceae, respectively (Fig. 6;Table S3). Other saprotrophic fungi were dominated by *Mortierella*. Under more fertile conditions, saprotrophic ascomycetes from the genus *Tetracladium*, and pathogenic fungi from the genus *Dactylonectria* were more abundant. Under less fertile conditions, saprotrophic basidiomycetes from the genera *Geminibasidium* and *Saitozyma* were more abundant (Fig. 6).

The first DCA axis identified a correlation between the fungal community composition and fertility and associated changes in potential enzyme activity and microbial respiration (Fig. 6); the second DCA axis revealed a correlation with mineral soil C : N ratios. Individual CCA analysis revealed fertility to explain 13.2% of the variance among the fungal community composition (total inertia was 1.026). Phenol oxidase, cellobiohydrolase, microbial respiration and soil C : N ratios were significant and explained 12.1, 9.1, 9.3 and 10.6% of the variance, respectively; other enzymes were not significant and are therefore not displayed. A stepwise backward variable selection (starting with all variables) revealed that fertility and soil C : N ratios together explained 21.5% of the variance.

### Discussion

Exploring feedback mechanisms between fertility and SOM turnover is critical to a better understanding of C and nutrient cycling in forest ecosystems. Here, we showed that proxies for SOM decomposition related to a shift in the soil fungal community composition along a fertility gradient in a temperate mountain forest of European beech.

Our first hypothesis - SOM decomposition will be positively related to fertility (Fig. 1b) - was strongly supported by our results: more fertile plots featured a shorter residence time of the organic layer and higher rates of microbial respiration, potential phenol oxidase and leucine aminopeptidase activities in the mineral soil (Fig. 4). The potential activity of most enzymes was also positively related to above-ground woody biomass of trees, indicating an indirect fertility effect via productivity (Table 1). This pattern was mirrored in lower organic layer C and N stocks (Fig. 3a,b) and lower mineral soil C and N concentrations at more fertile plots (Fig. S3). As stone content and fine soil bulk density showed opposing trends along the fertility gradient (Fig. S3), C and N stocks of the mineral soil (0-10 cm) were, however, similar across the gradient (Fig. 3d,e). Nevertheless, as tree biomass stocks and annual litter fall increased along with fertility (Fig. 2), the results supported a hypothesised, faster SOM turnover under more fertile conditions (Fig. 1b). Such a positive relationship between fertility and SOM decomposition is in line with previous studies from temperate and boreal forests (Ladegaard-Pedersen *et al.*, 2005; Kyaschenko *et al.*, 2017b, 2019). In addition to a better water supply and an inherently better mineral nutrient supply (e.g. potassium, magnesium) from the loamy subsoils (Figs 1a,S2), a faster SOM turnover and a related nutrient mobilisation point towards a positive feedback on ecosystem fertility, expressed in a higher above-ground woody biomass of trees and a modified composition of the herbal layer. We acknowledge that we could not confidently assess fertility effects on total soil C storage, as subsoils were only analysed at selected locations (Table S2). However, it is reasonable to speculate that loamy subsoils at more fertile plots store considerable amounts of C, which may additionally be stabilised by aggregate formation or association with mineral surfaces (Schmidt *et al.*, 2011; Lehmann & Kleber, 2015).

Our second hypothesis - fertility coincides with a shift in the soil fungal community composition that consequently influences SOM decomposition (Fig. 1b) - was also supported by our results: fungal community structure and total fungal DNA (as proxy for fungal biomass) changed along the gradient, with saprotrophic ascomycetes being relatively more abundant at more fertile plots (Figs 5,6). Moreover, the results indicate a strong control of total fungal DNA and saprotrophic ascomycetes on microbial respiration and potential phenol oxidase activity (Table 2), suggesting ascomycetes to be a prominent regulator of SOM decomposition in the studied mountain forest. Although saprotrophic ascomycetes are known to be less efficient decomposers than basidiomycetes, they can produce hydrolysing and oxidising enzymes that degrade SOM (Baldrian, 2006; Grinhut *et al.*, 2007; Eichlerová *et al.*, 2015; Kohler *et al.*, 2015). In particularly fungi from the groups of *Tetracladium* and *Hyaloscyphaceae* (higher abundance at more fertile plots; Fig. 6) were reported to have considerable decay capacities with respect to organic substrates (Abdel-Raheem, 1997; Boberg *et al.*, 2011; Anderson & Marvanova, 2020). By contrast, previous gradient studies in boreal pine and spruce forests reported a lower ascomycetes abundance and a higher basidiomycetes abundance under more fertile conditions; basidiomycetes were therefore suggested to be the principal regulator of SOM decomposition in these forest ecosystems (Sterkenburg *et al.*, 2015; Kyaschenko *et al.*, 2017b). In the mountain beech forest of this study, the abundance of saprotrophic basidiomycetes was, except for a few taxa (e.g. *Geminibasidium),* largely independent of fertility (Figs 5d,6).

Ratios between relative abundance of ectomycorrhizal fungi and saprotrophic ascomycetes were higher under less fertile conditions (Fig. 5h), suggesting a greater competition pressure of ectomycor- rhizal fungi on this saprotrophic guild. A negative correlation between fertility and mineral soil C : N ratios (Fig. 3f) might also point towards a higher ectomycorrhizal N foraging in mineral soils at the less fertile end of the gradient (Averill *et al.*, 2014; Fernandez & Kennedy, 2016; Smith & Wan, 2019). Particularly *Tomentella* and *Clavulina* were related to wider mineral soil C: N ratios (Fig. 6). In a recent study, fungi from the genus *Tomentella* were suggested to be strongly involved in the suppression of saprotrophic decomposition thereby being key for driving the ‘Gadgil effect’ (Fernandez *et al.*, 2020). However, neither microbial respiration nor potential phenol oxidase activity were related to relative ectomy- corrhizal abundance or its ratios to saprotrophic fungi (Tables S4,S5). An ectomycorrhizal suppression of SOM decomposition under less fertile conditions could therefore not clearly be identified. This contradicts previous indications for a ‘Gadgil effect’ along a boreal forest gradient (Kyaschenko *et al.*, 2017b). Slow SOM turnover rates in boreal coniferous forest can result in the build-up of thick organic layers and the establishment of perennial fungal communities with a high potential for interguild competition (Clemmensen *et al.*, 2015; Kyaschenko *et al.*, 2017a). Temperate forests have a generally faster SOM turnover compared with boreal forests (Wang *et al.*, 2018). Moreover, ectomycorrhizal constraints on decomposition were shown to be present only when substrate quality is low (Smith & Wan, 2019; Fernandez *et al.*, 2020). It is therefore likely that the studied temperate beech forest features less (intense) competitive interaction between fungal guilds, potentially related to a more ephemeral fungal community and/or a higher substrate quality compared with boreal coniferous forests. However, we cannot rule out that saprotrophic decomposition was suppressed by ecto- mycorrhizal fungi also at the studied forest site.

Priming might be a process underlying higher SOM decomposition under more fertile conditions. Rhizodeposition can account for up to a third of the photosynthetically fixed C and depends on stand productivity and root biomass (Jones *et al.*, 2009; Phillips *et al.*, 2011; Liese *et al.*, 2018). Moreover, priming is positively affected by above-ground biomass and tree productivity (Hoosbeek *et al.*, 2004; Huo *et al.*, 2017). While we did not measure rhizodeposition, it is reasonable to speculate that the greater fine root biomass at more fertile plots is indicative of more labile C being released to the rhizosphere, thereby potentially stimulating saprotrophic decomposition. This assumption is backed up by earlier findings, where tree girdling, root trenching and clearcutting in temperate oak-beech, oak, and spruce forests decreased litter decomposition and soil enzymatic activity, indicating that rhizosphere C input primes SOM decomposition (Brzostek *et al.*, 2015; Kohout *et al.*, 2018; Fernandez *et al.*, 2020). In addition to priming by the input of labile C exudates from roots and ectomycorrhizas, decomposition might also be stimulated by the input of dead fine roots and fungal mycelium (Brabcová *et al.*, 2016; Fernandez & Kennedy, 2016; Frey, 2019).

A positive relationship between fertility and SOM decomposition may also be linked to other abiotic and biotic factors. Soil temperature and moisture, for example, are known to strongly control microbial activity (Moyano *et al*., 2012; Mayer *et al.*, 2017b). Here, fertile conditions were associated with greater soil water availability, as assessed by Ellenberg indicator values (Fig. S2); measured volumetric water content in the topsoil was, however, constant along the fertility gradient (Fig. S3). Soil temperature was even slightly lower at more fertile plots (Fig. S3), potentially due to greater shading by canopies. This would suggest microclimatic differences in the upper soil horizons to be of minor importance for SOM decomposition along the fertility gradient. An enhanced water supply to trees, however, might again result in a positive feedback on SOM decomposition via increased productivity and a related priming effect (Hoosbeek *et al*., 2004; Huo *et al*., 2017). Other microbial and faunal groups (e.g. bacteria, earthworms) and potential interactions with soil fungi may also be of great relevance for SOM decomposition (Baldrian, 2017). Particularly earthworms could play a crucial role in litter decay at fertile plots with loamy subsoils (Barthod *et al*., 2020). Moreover, high quality litter from geophytes (e.g. *Allium ursinum;* Table S1) (Jandl *et al.*, 1997; Prescott, 2010) and higher N mineralisation rates at fertile plots could be important factors contributing to a positive fertility feedback (Norris *et al.*, 2013).

Taken together, our results indicated a strong interdependency between soil fertility, SOM decomposition and soil fungal community composition in the studied mountain forest ecosystem. The results furthermore show a fertility-SOM decomposition feedback mechanism, which is mediated by soil fungi to a significant extent. We did not find a clear indication that ectomycorrhizal fungi constrained saprotrophs along the gradient, but we suggest that SOM decomposition at more fertile plots was stimulated by rhizosphere priming. Moreover, we suggest that a higher SOM decomposition and associated nutrient mobilisation under fertile conditions result in a positive feedback on tree growth. As our conclusions are based on correlation analyses, manipulation experiments (e.g. tree girdling) and/or additional in-depth measurements (e.g. on rhizodeposition, interaction of fungi with other soil organisms) incl. deeper soil horizons are needed to further improve our mechanistic understanding of the important yet complex influence of soil fungal interactions on the carbon and nutrient cycle.

## Supplementary Material

Fig. S1, Fig. S2, Fig. S3, Fig. S4, Methods S1, Methods S2

Table S1

Table S2

Table S3

Table S4

Table S5

Supporting Information

## Figures and Tables

**Table 1:** Relationship between above-ground woody biomass of trees (m ^3^ ha ^1^) and microbial respiration, and potential soil enzyme activities, determined for the mineral soil (0-10 cm) of a fertility gradient in a beech dominated mountain forest.

Variable	Unit	Slope	P-value	R^2^
Microbial respiration	g C m^-2^ d^-1^	(+)	0.008	0.41
Phenol oxidase	mol m^-2^ h^-1^	(+)	0.006	0.43
ß-Glucosidase	mmol m^-2^ h^-1^	(+)	0.035	0.28
Cellobiohydrolase	mmol m^-2^ h^-1^	(+)	0.136	0.15
ß-Xylosidase	mmol m^-2^ h^-1^	(+)	0.037	0.28
Leucine aminopeptidase	mmol m^-2^ h^-1^	(+)	0.001	0.56
N-Acetylglucosaminidase	mmol m^-2^ h^-1^	(+)	0.001	0.54
Acid phosphatase	mmol m^-2^ h^-1^	(+)	0.005	0.44

Test statistics of linear regression models are given; directions of slope coefficients are indicated (*n* = 16).

**Table 2:** Relationship between relative abundance of saprotrophic ascomycetes (%) and total fungal DNA in soil (μg m ^2^) and microbial respiration, and potential enzyme activities, determined for the mineral soil (0-10 cm) of a fertility gradient in a mountain beech forest.

		Saprotrophic ascomycetes			Total fungal DNA		
Variable	Unit	Slope	P-value	R^2^	Slope	P-value	R^2^
Microbial respiration	g C m^—2^ d^—1^	(+)	0.041	0.27	(+)	0.001	0.53
Phenol oxidase	mol m^—2^ h^—1^	(+)	0.002	0.5	(+)	< 0.001	0.85
ß-Glucosidase	mmol m^—2^ h^—1^	(+)	0.052	0.24	(+)	0.003	0.48
Cellobiohydrolase	mmol m^—2^ h^—1^	(+)	0.064	0.22	(+)	0.04	0.27
ß-Xylosidase	mmol m^—2^ h^—1^	(+)	0.054	0.24	(+)	0.004	0.46
Leucine aminopeptidase	mmol m^—2^ h^—1^	(+)	0.124	0.16	(+)	0.001	0.56
N-Acetyl-glucosaminidase	mmol m^—2^ h^—1^	(+)	0.458	0.04	(+)	0.008	0.41
Acid phosphatase	mmol m^—2^ h^—1^	(+)	0.266	0.09	(+)	< 0.001	0.63

Given are test statistics of linear regression models; directions of slope coefficients are indicated (n = 16). Information on correlations with other fungal guilds is given in Supporting Information Table S4.

## Data Availability

All data used for the analyses of this study are publicly accessible. Sequencing and associated data have been deposited at NCBI BioProject PRJNA521677, BioSamples SAMN12582230- SAMN12582341 and GenBank accession numbers MK626959- MK627467.

## References

[R1] Abdel-Raheem A (1997). Laccase activity of lignicolous aquatic hyphomycetes isolated from the River Nile in Egypt. Mycopathologia.

[R2] Anderson JL, Marvanová L (2020). Broad geographical and ecological diversity from similar genomic toolkits in the ascomycete genus Tetracladium. bioRxiv.

[R3] Averill C, Hawkes CV (2016). Ectomycorrhizal fungi slow soil carbon cycling. Ecology Letters.

[R4] Averill C, Turner BL, Finzi AC (2014). Mycorrhiza-mediated competition between plants and decomposers drives soil carbon storage. Nature.

[R5] Baldrian P (2006). Fungal laccases-occurrence and properties. FEMS Microbiology Reviews.

[R6] Baldrian P, Boddy L, Frankland JC, van West P (2008). Enzymes of saprotrophic basidiomycetes. Ecology of saprotrophic basidiomycetes.

[R7] Baldrian P (2009). Ectomycorrhizal fungi and their enzymes in soils: is there enough evidence for their role as facultative soil saprotrophs?. Oecologia.

[R8] Baldrian P (2017). Forest microbiome: diversity, complexity and dynamics. FEMS Microbiology Reviews.

[R9] Barthod J, Dignac MF, Le Mer G, Bottinelli N, Watteau F, Kügel-Knabner I, Rumpel C (2020). How do earthworms affect organic matter decomposition in the presence of clay-sized minerals?. Soil Biology and Biochemistry.

[R10] Bastida F, García C, Fierer N, Eldridge DJ, Bowker MA, Abades S, Alfaro FD, Asefaw Berhe A, Cutler NA, Gallardo A (2019). Global ecological predictors of the soil priming effect. Nature Communications.

[R11] Berger TW, Untersteiner H, Toplitzer M, Neubauer C (2009). Nutrient fluxes in pure and mixed stands of spruce *Picea abies* and beech *Fagus sylvatica*. Plant and Soil.

[R12] Binkley D, Fisher RF (2013). Ecology and management of forest soils.

[R13] Boberg JB, Ihrmark K, Lindahl BD (2011). Decomposing capacity of fungi commonly detected in *Pinus sylvestris* needle litter. Fungal Ecology.

[R14] Brabcová V, Novakova M, Davidová A, Baldrian P (2016). Dead fungal mycelium in forest soil represents a decomposition hotspot and a habitat for a specific microbial community. New Phytologist.

[R15] Brzostek ER, Dragoni D, Brown ZA, Phillips RP (2015). Mycorrhizal type determines the magnitude and direction of root-induced changes in decomposition in a temperate forest. New Phytologist.

[R16] Chapin FS, Matson PA, Mooney HA (2002). Principles of terrestrial ecosystem ecology.

[R17] Clemmensen KE, Finlay RD, Dahlberg A, Stenlid J, Wardle DA, Lindahl BD (2015). Carbon sequestration is related to mycorrhizal fungal community shifts during long-term succession in boreal forests. New Phytologist.

[R18] Crowther TW, Van den Hoogen J, Wan J, Mayes MA, Keiser A, Mo L, Averill C, Maynard DS (2019). The global soil community and its influence on biogeochemistry. Science.

[R19] Deltedesco E, Keiblinger KM, Piepho H-P, Antonielli L, Potsch EM, Zechmeister-Boltenstern S, Gorfer M (2020). Soil microbial community structure and function mainly respond to indirect effects in a multifactorial climate manipulation experiment. SoilBiology and Biochemistry.

[R20] Eichlerova I, Homolka L, žifcákova L, Lisa L, Dobiášová P, Baldrian P (2015). Enzymatic systems involved in decomposition reflects the ecology and taxonomy of saprotrophic fungi. Fungal Ecology.

[R21] Ellenberg H, Weber HE, Düll R, Wirth V, Werner W, Paulißen D (1992). Zeigerwertevon Pflanzen in Mitteleuropa. Scripta Geobotanica.

[R22] Feinstein LM, Sul WJ, Blackwood CB (2009). Assessment of bias associated with incomplete extraction of microbial DNA from soil. Applied and Environment Microbiology.

[R23] Fernandez CW, Kennedy PG (2016). Revisiting the ‘Gadgil effect’: do interguild fungal interactions control carbon cycling in forest soils?. New Phytologist.

[R24] Fernandez CW, See CR, Kennedy PG (2020). Decelerated carbon cycling by ectomycorrhizal fungi is controlled by substrate quality and community composition. New Phytologist.

[R25] Fontaine S, Barot S, Barre P, Bdioui N, Mary B, Rumpel C (2007). Stability of organic carbon in deep soil layers controlled by fresh carbon supply. Nature.

[R26] Frey SD (2019). Mycorrhizal fungi as mediators of soil organic matter dynamics. Annual Review of Ecology, Evolution, and Systematics.

[R27] Gadgil RL, Gadgil PD (1971). Mycorrhiza and litter decomposition. Nature.

[R28] German DP, Weintraub MN, Grandy AS, Lauber CL, Rinkes ZL, Allison SD (2011). Optimization of hydrolytic and oxidative enzyme methods for ecosystem studies. Soil Biology and Biochemistry.

[R29] Gorfer M, Mayer M, Berger H, Rewald B, Tallian C, Matthews B, Sanden H, Katzensteiner K, Godbold DL (2021). High fungal diversity but low seasonal dynamics and ectomycorrhizal abundance in a Mountain Beech forest. Microbial Ecology.

[R30] Grinhut T, Hadar Y, Chen Y (2007). Degradation and transformation of humic substances by saprotrophic fungi: processes and mechanisms. Fungal Biology Reviews.

[R31] Guenet B, Camino-Serrano M, Ciais P, Tifafi M, Maignan F, Soong JL, Janssens IA (2018). Impact of priming on global soil carbon stocks. Global Change Biology.

[R32] Hansson K, Laclau J-P, Saint-André L, Mareschal L, van der Heijden G, Nys C, Nicolas M, Ranger J, Legout A (2020). Chemical fertility of forest ecosystems. Part 1: Common soil chemical analyses were poor predictors of stand productivity across a wide range of acidic forest soils. Forest Ecology and Management.

[R33] Hofstetter V, Buyck B, Eyssartier G, Schnee S, Gindro K (2019). The unbearable lightness of sequenced-based identification. Fungal Diversity.

[R34] Hoosbeek MR, Lukac M, van Dam D, Godbold DL, Velthorst EJ, Biondi FA, Peressotti A, Cotrufo MF, de Angelis P, Scarascia-Mugnozza G (2004). More new carbon in the mineral soil of a poplar plantation under Free Air Carbon Enrichment (POPFACE): cause of increased priming effect?. Global Biogeochemical Cycles.

[R35] Huo C, Luo Y, Cheng W (2017). Rhizosphere priming effect: a meta-analysis. Soil Biology and Biochemistry.

[R36] Jandl R, Kopeszki H, Glatzel G (1997). Effect of a dense *Allium ursinum* (L.) ground cover on nutrient dynamics and mesofauna of a *Fagus sylvatica* (L.) woodland. Plant and Soil.

[R37] Jones DL, Nguyen C, Finlay RD (2009). Carbon flow in the rhizosphere: carbon trading at the soil-root interface. Plant and Soil.

[R38] Keiblinger KM, Schneider M, Gorfer M, Paumann M, Deltedesco E, Berger H, Jochlinger L, Mentler A, Zechmeister-Boltenstern S, Soja G (2018). Assessment of Cu applications in two contrasting soils — effects on soil microbial activity and the fungal community structure. Ecotoxicology.

[R39] Kobler J, Jandl R, Dirnböck T, Mirtl M, Schindlbacher A (2015). Effects of stand patchiness due to windthrow and bark beetle abatement measures on soil CO_2_ efflux and net ecosystem productivity of a managed temperate mountain forest. European Journal of Forest Research.

[R40] Kohler A, Kuo A, Nagy LG, Morin E, Barry KW, Buscot F, Canbäck B, Choi C, Cichocki N, Clum A (2015). Convergent losses of decay mechanisms and rapid turnover of symbiosis genes in mycorrhizal mutualists. Nature Genetics.

[R41] Kohout P, Charvátová M, Štursová M, Mašmová T, Tomၡovský M, Baldrian P (2018). Clearcutting alters decomposition processes and initiates complex restructuring of fungal communities in soil and tree roots. The ISME Journal.

[R42] Kõljalg U, Nilsson RH, Abarenkov K, Tedersoo L, Taylor AFS, Bahram M, Bates ST, Bruns TD, Bengtsson-Palme J, Callaghan TM (2013). Towards a unified paradigm for sequence-based identification of fungi. Molecular Ecology.

[R43] Kyaschenko J, Clemmensen KE, Hagenbo A, Karltun E, Lindahl BD (2017a). Shift in fungal communities and associated enzyme activities along an age gradient of managed *Pinus sylvestris* stands. The ISME Journal.

[R44] Kyaschenko J, Clemmensen KE, Karltun E, Lindahl BD (2017b). Below-ground organic matter accumulation along a boreal forest fertility gradient relates to guild interaction within fungal communities. Ecology Letters.

[R45] Kyaschenko J, Ovaskainen O, Ekblad A, Hagenbo A, Karltun E, Clemmensen KE, Lindahl BD (2019). Soil fertility in boreal forest relates to root-driven nitrogen retention and carbon sequestration in the mor layer. New Phytologist.

[R46] Ladegaard-Pedersen P, Elberling B, Vesterdal L (2005). Soil carbon stocks, mineralization rates, and CO_2_effluxes under 10 tree species on contrasting soil types. Canadian Journal of Forest Research.

[R47] Lehmann J, Kleber M (2015). The contentious nature of soil organic matter. Nature.

[R48] Liese R, Lübbe T, Albers NW, Meier IC (2018). The mycorrhizal type governs root exudation and nitrogen uptake of temperate tree species. Tree Physiology.

[R49] Lindahl BD, Tunlid A (2015). Ectomycorrhizal fungi — potential organic matter decomposers, yet not saprotrophs. New Phytologist.

[R50] Liu CM, Kachur S, Dwan MG, Abraham AG, Aziz M, Hsueh P-R, Huang Y-T, Busch JD, Lamit LJ, Gehring CA (2012). FungiQuant: a broad-coverage fungal quantitative real-time PCR assay. BMC Microbiology.

[R51] Marx MC, Wood M, Jarvis SC (2001). A microplate fluorimetric assay for the study of enzyme diversity in soils. Soil Biology and Biochemistry.

[R52] Mayer M, Matthews B, Rosinger C, Sandén H, Godbold DL, Katzensteiner K (2017a). Tree regeneration retards decomposition in a temperate mountain soil after forest gap disturbance. Soil Biology and Biochemistry.

[R53] Mayer M, Sandén H, Rewald B, Godbold DL, Katzensteiner K (2017b). Increase in heterotrophic soil respiration by temperature drives decline in soil organic carbon stocks after forest windthrow in a mountainous ecosystem. Functional Ecology.

[R54] Moyano Fe, Vasilyeva N, Bouckaert L, Cook F, Craine J, Curiel Yuste J, Don A, Epron D, Formanek P, Franzluebbers A (2012). The moisture response of soil heterotrophic respiration: interaction with soil properties. Biogeosciences.

[R55] Norris MD, Avis PG, Reich PB, Hobbie SE (2013). Positive feedbacks between decomposition and soil nitrogen availability along fertility gradients. Plant and Soil.

[R56] Oksanen J, Blanchet FG, Kindt R, Legendre P, Minichin PR, O’Hara RB, Simpson GL, Solymos P, Stevens MHH, Wagner H (2016). vegan: Community ecology package.

[R57] O NORM L 1084 (1999). Chemical analyses of soils - determination of carbonate taking into account air pressure and temperature.

[R58] Orwin KH, Kirschbaum MU, St John MG, Dickie IA (2011). Organic nutrient uptake by mycorrhizal fungi enhances ecosystem carbon storage: a model-based assessment. Ecology Letters.

[R59] Paliy O, Shankar V (2016). Application of multivariate statistical techniques in microbial ecology. Molecular Ecology.

[R60] Phillips RP, Finzi AC, Bernhardt ES (2011). Enhanced root exudation induces microbial feedbacks to N cycling in a pine forest under long-term CO_2_fumigation. Ecology Letters.

[R61] Phillips RP, Meier IC, Bernhardt ES, Grandy AS, Wickings K, Finzi AC (2012). Roots and fungi accelerate carbon and nitrogen cycling in forests exposed to elevated CO2. Ecology Letters.

[R62] Pinheiro JC, Bates DM, DebRoy S, Sarkar D, R Core Team (2014). nlme: Linear and nonlinear mixed effects models.

[R63] Prescott CE (2010). Litter decomposition: what controls it and how can we alter it to sequester more carbon in forest soils?. Biogeochemistry.

[R64] R Core Team (2017). R: a language and environment for statistical computing.

[R65] Read D, Perez-Moreno J (2003). Mycorrhizas and nutrient cycling in ecosystems-a journey towards relevance?. New Phytologist.

[R66] Reichstein M, Bednorz F, Broll G, Kätterer T (2000). Temperature dependence of carbon mineralisation: conclusions from a long-term incubation of subalpine soil samples. Soil Biology and Biochemistry.

[R67] Rognes T, Flouri T, Nichols B, Quince C, Mahe F (2016). VSEARCH: a versatile open source tool for metagenomics. PeerJ.

[R68] Sariyildiz T, Anderson JM (2003). Interactions between litter quality, decomposition and soil fertility: a laboratory study. Soil Biology and Biochemistry.

[R69] Schindlbacher A, Schnecker J, Takriti M, Borken W, Wanek W (2015). Microbial physiology and soil CO2 efflux after 9 years of soil warming in a temperate forest - no indications for thermal adaptations. Global Change Biology.

[R70] Schmidt MWI, Torn MS, Abiven S, Dittmar T, Guggenberger G, Janssens IA, Kleber M, Kogel-Knabner I, Lehmann J, Manning DAC (2011). Persistence of soil organic matter as an ecosystem property. Nature.

[R71] Schneider T, Keiblinger KM, Schmid E, Sterflinger-Gleixner K, Ellersdorfer G, Roschitzki B, Richter A, Eberl L, Zechmeister-Boltenstern S, Riedel K (2012). Who is who in litter decomposition? Metaproteomics reveals major microbial players and their biogeochemical functions. The ISME Journal.

[R72] Smith GR, Wan J (2019). Resource-ratio theory predicts mycorrhizal control of litter decomposition. New Phytologist.

[R73] Sterkenburg E, Bahr A, Brandström Durling M, Clemmensen KE, Lindahl BD (2015). Changes in fungal communities along a boreal forest soil fertility gradient. New Phytologist.

[R74] Sterkenburg E, Clemmensen KE, Ekblad A, Finlay RD, Lindahl BD (2018). Contrasting effects of ectomycorrhizal fungi on early and late stage decomposition in a boreal forest. The ISME Journal.

[R75] Tedersoo L, Anslan S, Bahram M, Põme S, Riit T, Liiv I, Kõljalg U, Kisand V, Nilsson H, Hildebrand F (2015). Shotgun metagenomes and multiple primer pair-barcode combinations of amplicons reveal biases in metabarcoding analyses of fungi. MycoKeys.

[R76] Unterwurzacher V, Pogner C, Berger H, Strauss J, Strauss-Goller S, Gorfer M (2018). Validation of a quantitative PCR based detection system for indoor mold exposure assessment in bioaerosols. Environmental Science: Processes & Impacts.

[R77] Van Der Heijden MG, Martin FM, Selosse MA, Sanders IR (2015). Mycorrhizal ecology and evolution: the past, the present, and the future. New Phytologist.

[R78] Vesterdal L, Raulund-Rasmussen K (1998). Forest floor chemistry under seven tree species along a soil fertility gradient. Canadian Journal of Forest Research.

[R79] Vicca S, Luyssaert S, Peñuelas J, Campioli M, Chapin Fs, Ciais P, Heinemeyer A, Högberg P, Kutsch Wl, Law Be (2012). Fertile forests produce biomass more efficiently. Ecology Letters.

[R80] Wang J, Sun J, Xia J, He N, Li M, Niu S (2018). Soil and vegetation carbon turnover times from tropical to boreal forests. Functional Ecology.

[R81] White TJ, Bruns T, Lee S, Taylor J (1990). Amplification and direct sequencing of fungal ribosomal RNA genes for phylogenetics. PCR Protocols: A Guide To Methods and Applications.

[R82] Wutzler T, Wirth C, Schumacher J (2008). Generic biomass functions for Common beech *Fagus sylvatica* in Central Europe: predictions and components of uncertainty. Canadian Journal of Forest Research.

[R83] Zak DR, Pellitier PT, Argiroff WA, Castillo B, James TY, Nave LE, Averill C, Beidler KV, Bhatnagar J, Blesh J (2019). Exploring the role of ectomycorrhizal fungi in soil carbon dynamics. New Phytologist.

[R84] Zuur AF, Ieno EN, Walker NJ, Saveliev AA, Smith GM (2009). Mixed effects models and extensions in ecology with R.

