# Supplementary material for: Soil fertility relates to fungal-mediated decomposition and organic matter turnover in a temperate mountain forest
Source: New Phytol. Author manuscript; Available in PMC 2021 Jul 1. (PMC7611052; doi:10.1111/nph.17421)
Supplement: Fig. S1, Fig. S2, Fig. S3, Fig. S4, Methods S1, Methods S2 [file EMS126083-supplement-Fig__S1__Fig__S2__Fig__S3__Fig__S4__Methods_S1__Methods_S2.pdf]

New Phytologist

***New Phytologist* Supporting Information**

Article title: Soil fertility relates to fungal-mediated decomposition and organic matter turnover in a temperate mountain forest

Authors: Mathias Mayer, Boris Rewald, Bradley Matthews, Hans Sandén, Christoph Rosinger, Klaus Katzensteiner, Markus Gorfer, Harald Berger, Claudia Tallian, Torsten W. Berger, Douglas L. Godbold

Article acceptance date: 08 April 2021

The following Supporting Information is available for this article:

Fig. S1 Layout of the 16 plots along the fertility gradient.

Fig. S2 Principal component analysis (PCA) analysing Ellenberg indicator values of vascular plants of the ground vegetation layer.

Fig. S3 Relationship between fertility index and mineral soil organic C concentration, N concentration, inorganic C concentration, pH, bulk density, stone content, moisture, and temperature, respectively.

Fig. S4 Relationship between fertility index and selected fungal guild ratios.

Table S1 List of vascular plant species in the ground vegetation layer and their Ellenberg indicator values.

Table S2 Total mineral soil organic C and N stocks.

Table S3 List of fungal taxonomic groups including abundance and lifestyle/guild.

Table S4 Relationship between relative abundance of selected soil fungal guilds, guild ratios and microbial respiration, and potential enzyme activities in mineral soil.

Table S5 Relationship between relative abundance of selected soil fungal guilds and microbial respiration, and potential enzyme activities per g soil C.

Methods S1 Determination of total mineral soil organic carbon and nitrogen stocks.

Methods S2 Calculation of integrative fertility index based on Ellenberg indicator values.

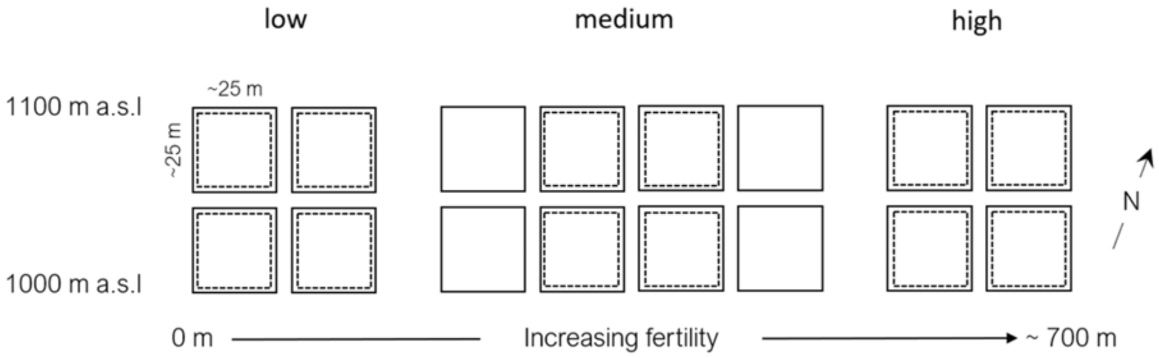

Fig. S1 Layout of the 16 plots along the fertility gradient in a mountain forest dominated by European beech. Solid line rectangles depict plots for mineral soil analyses. Dotted line rectangles depict plots for organic layer analyses. Fertility levels (low, medium, high) associated with litter traps are depicted. Height above sea level (m a.s.l.), approx. length of the fertility gradient (m) and exposition of plots are indicated; drawing not at scale.

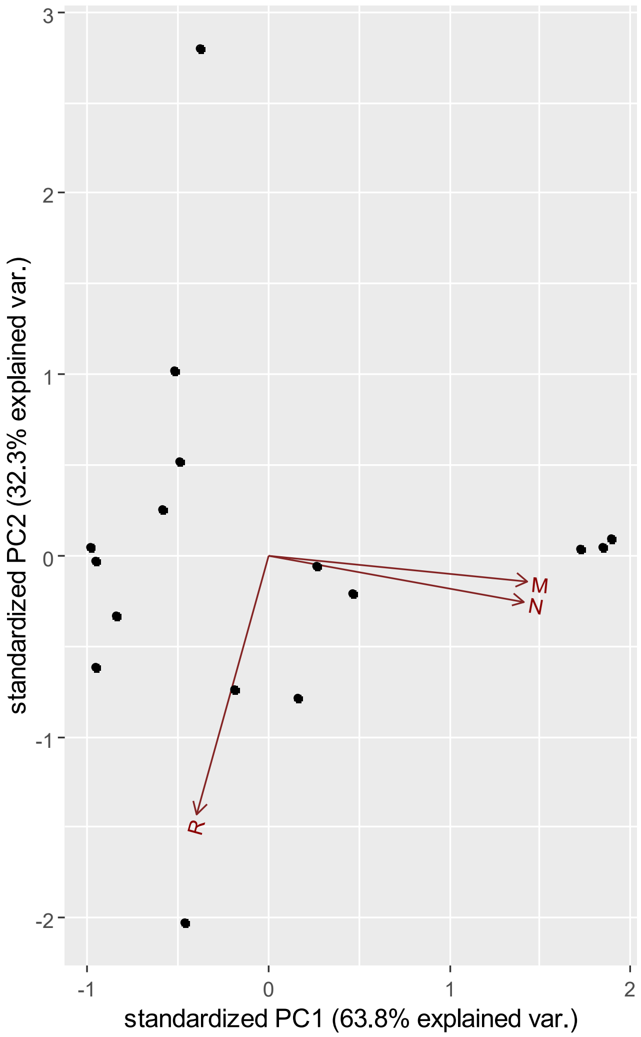

Fig. S2 Principal component analysis (PCA) analysing Ellenberg indicator values of vascular plants of the ground vegetation layer along a fertility gradient in a mountain forest of European beech. The scores of the first PCA axis were used as a fertility index; see Supplementary Information for details (Methods S2). Abbreviations of Ellenberg indicator values: nutrients (N), moisture (M), soil reaction (R).

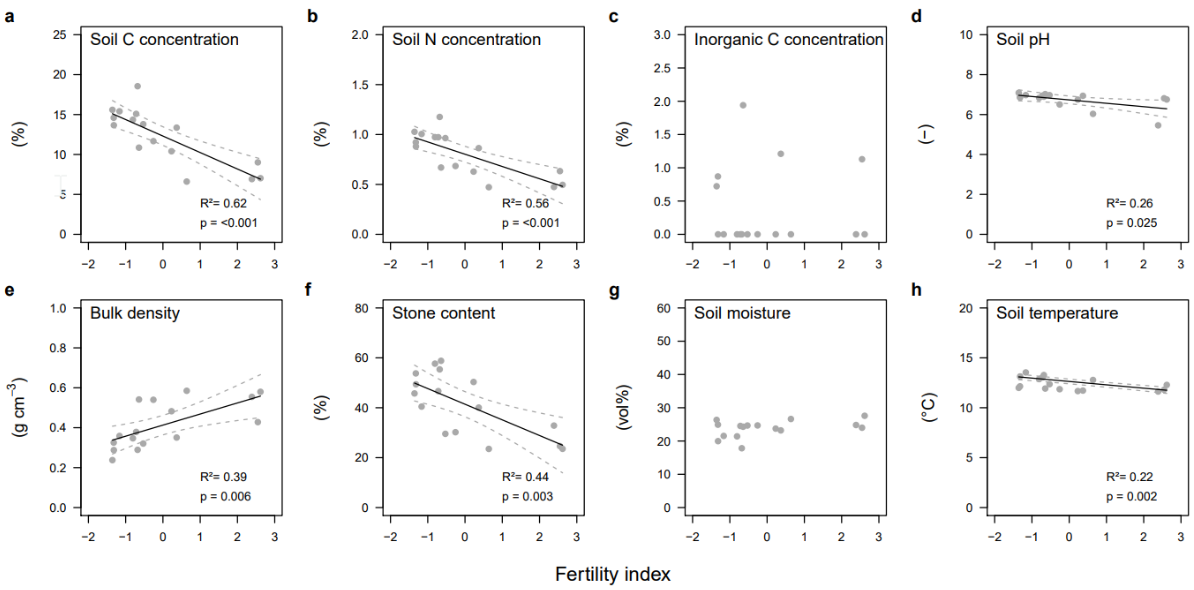

Fig. S3 Relationship between fertility index and mineral soil (0-10 cm) (a) C concentration, (b) N concentration, (c) inorganic C concentration, (d) pH, (e) fine soil bulk density, (f) stone content (given in percentage soil volume), and (g) soil moisture in 0-7 cm depth (given in volumetric soil water content), and (h) soil temperature in 5 cm depth, respectively. The fertility index is based on the first axis of a principal component analysis of Ellenberg indicator values for vascular plants (Fig. S2). Given are test statistics of significant linear regression models (n = 16); solid lines and dashed grey lines show fitted models and 95% confidence intervals, respectively.

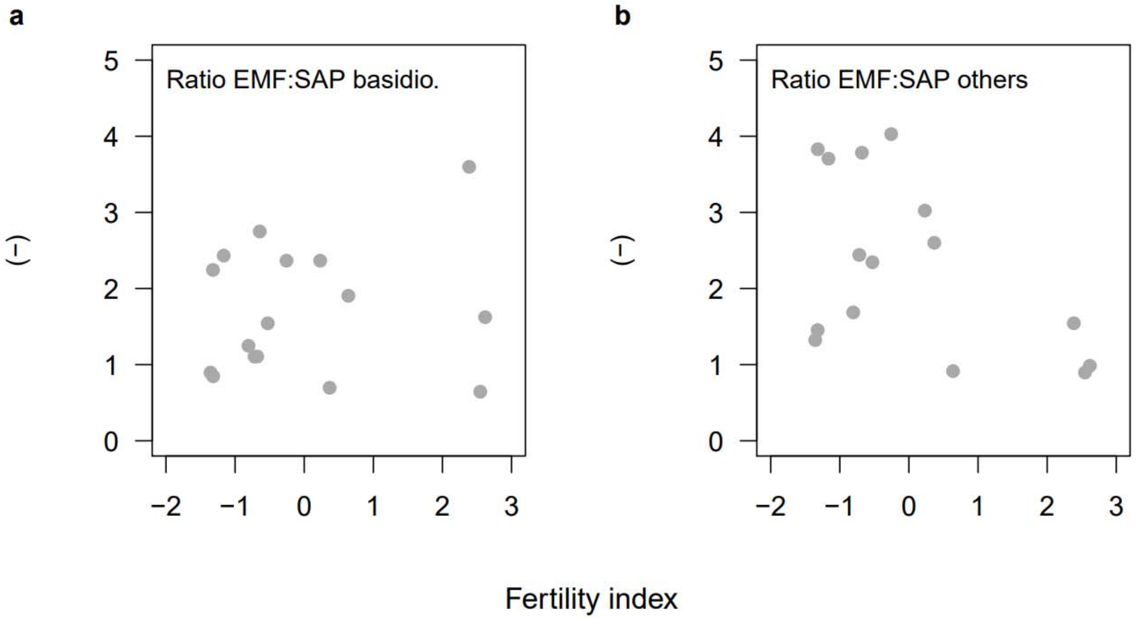

Fig. S4 Relationship between fertility index and the relative abundance ratios of (a) ectomycorrhizal fungi (EMF) to saprotrophic (SAP) basidiomycetes, and (b) ectomycorrhizal fungi to other saprotrophic fungi. The fertility index is based on the first axis of a principal component analysis of Ellenberg indicator values for vascular plants (Fig. S2).

Table S1 List of vascular plant species in the ground vegetation layer and their Ellenberg indicator values at a fertility gradient in a mountain forest of European beech. Abbreviations for Ellenberg indicator values: nutrients (N), moisture (M), and soil reaction (R). (see separate file)

Table S2 Table S2 Total mineral soil organic C and N stocks determined for soil pits along a fertility gradient in a mountain beech forest. Given are mean values and standard error of the mean (n=4). Note: for soil depth 30-40 cm only one sample has been taken. (see separate file)

Table S3 List of fungal taxonomic groups including abundance and lifestyle/guild detected on a fertility gradient in a mountain forest of European beech. See text for details. (see separate file)

Table S4 Relationship between relative abundance of selected soil fungal guilds, guild relative abundance ratios, microbial respiration, and potential enzyme activities determined for a fertility gradient in a mountain beech forest. Given are test statistics of linear regression models; directions of slope coefficients are indicated (n = 16). (see separate file)

Table S5 Relationship between relative abundance of soil fungal guilds and microbial respiration, and potential enzyme activities, determined for a fertility gradient in a mountain beech forest. Given are test statistics of linear regression models; directions of slope coefficients are indicated (n = 16). Respiration and enzyme activities are expressed per g soil carbon (C). (see separate file)

See the Supporting MS Excel files for associated data in separate file sheets labelled Table S1-5.

Methods S1 Determination of total mineral soil organic carbon and nitrogen stocks.

To determine total mineral soil organic carbon (C) and nitrogen (N) stocks, large soil pits (~80 × 80 cm surface area) were dug down to bedrock at four additional locations along the fertility gradient. Samples were taken from mineral soil in four different depths (0-10, 10-20, 20-30, and 30-40 cm) if total depth allowed it. Stone content was estimated visually for each horizon using a grid frame (10 × 10 cm). Three soil samples were taken per horizon using steel cylinders (250 cm^3). Soil samples taken from large soil pits were pooled per pit and horizon and sieved (2 mm). In the laboratory, total C and N concentrations were analysed on 300 mg subsamples using a TruSpec CHN analyser (Leco, St Joseph, MI, USA); subsamples were dried (105°C, 24 hours) and ground prior to analysis (Pulverisette 5; Fritsch, Germany). Inorganic C content of subsamples was determined by the Scheibler method (ÖNORM L 1084, 1999). Organic soil C concentration was calculated as the difference of inorganic and total C concentration. Soil bulk density was determined, and total soil organic C and N stocks were calculated for each horizon (Table S2).

Methods S2 Calculation of integrative fertility index based on Ellenberg indicator values.

Soil fertility can be defined by biological- (e.g. decomposer activity), chemical- (e.g. nutrient availability, buffer capacity), and physical properties (e.g. water availability) that effect plant growth and their production (Hansson *et al.*, 2020). As there are no straightforward methods for quantifying plant available resources ('fertility'), the use of bio-indicator values as proxies is attractive (Diekmann, 2003; Mellert & Ewald, 2014). Ellenberg indicator values (EIV's) are frequently used as indicator values in vegetation science ((Ellenberg *et al.*, 1992; Ellenberg & Leuschner, 2010; Bartelheimer & Poschlod, 2016); in particular, the plant community-averaged EIV 'N' (nutrient values) is used as proxy for the availability of the macronutrients nitrogen, phosphorus, and potassium (Mellert and Ewald (2014), Bartelheimer and Poschlod (2016) and references within). As EIV 'R'(reaction values) approximate soil acidity (pH) and have been found to (partially) indicate the availability of P, Fe, Ca, Mg on calcareous soils (Zohlen & Tyler, 2004;

Ewald, 2009; Bartelheimer & Poschlod, 2016)—'R' can be regarded as a complementary candidate for assessing the nutrient availability. As nutrient availability ultimately depends on water availability—as nutrient solutions are taken up by roots—the EIV 'M' (soil moisture) is of additional key relevance. Taking this into account, we calculated an integrative 'fertility index'—based on the three EIV's 'N', 'M' and 'R'. Vascular plant species of the ground vegetation layer as determined during a vegetation survey were assigned EIV's (Table S1) and cover-weighted mean values were calculated per plot. The survey was limited to vascular species of the herbal layer, as bryophytes were found not to improve the usability of EIV in mountain forest stands (Ewald, 2009). The respective mean values of 'N', 'M', and 'R' per plot were subsequently analyzed by means of principal component analysis (PCA; Fig. S2). The scores of the first PCA axis were used as a 'fertility index'. The fertility index explained 63.8% of the variation among Ellenberg indicator values of the plant community composition at each subplot (Fig. S2). A similar approach, using PCA axis 1 as a fertility index but based on the variables soil N content, pH value, and litter C:N ratio, was recently successfully applied by Kyaschenko *et al.* (2017). To improve readability, the fertility index is referred to as 'fertility' throughout the manuscript.

References

Bartelheimer M, Poschlod P. 2016. Functional characterizations of Ellenberg indicator values – a review on ecophysiological determinants. *Functional Ecology* **30**(4): 506-516.

Diekmann M. 2003. Species indicator values as an important tool in applied plant ecology–a review. *Basic and applied ecology* **4**(6): 493-506.

Ellenberg H, Leuschner C. 2010. *Vegetation Mitteleuropas mit den Alpen*. Stuttgart: Ulmer.

Ellenberg H, Weber HE, Düll R, Wirth V, Werner W, Paulißen D. 1992. Zeigerwerte von Pflanzen in Mitteleuropa. *Scripta Geobotanica* **18**: 1-258.

Ewald J. 2009. Epigeic bryophytes do not improve bioindication by Ellenberg values in mountain forests. *Basic and applied ecology* **10**(5): 420-426.

Hansson K, Laclau J-P, Saint-André L, Mareschal L, van der Heijden G, Nys C, Nicolas M, Ranger J, Legout A. 2020. Chemical fertility of forest ecosystems. Part 1: Common soil chemical analyses were poor predictors of stand productivity across a wide range of acidic forest soils. *Forest Ecology and Management* **461**: 117843.

Kyaschenko J, Clemmensen KE, Karltun E, Lindahl BD. 2017. Below-ground organic matter accumulation along a boreal forest fertility gradient relates to guild interaction within fungal communities. *Ecology Letters* **20**(12): 1546-1555.

Mellert KH, Ewald J. 2014. Nutrient limitation and site-related growth potential of Norway spruce (Picea abies [L.] Karst) in the Bavarian Alps. *European Journal of Forest Research* **133**(3): 433-451.

ÖNORM L 1084 1999. Chemical analyses of soils - Determination of carbonate taking into account air pressure and temperature. Vienna, Austria: Austrian Standards Institute.

Zohlen A, Tyler G. 2004. Soluble inorganic tissue phosphorus and calcicole–calcifuge behaviour of plants. *Annals of Botany* **94**(3): 427-432.
