# Supplementary material for: Soil fertility relates to fungal-mediated decomposition and organic matter turnover in a temperate mountain forest
Source: New Phytol. Author manuscript; Available in PMC 2021 Jul 1. (PMC7611052; doi:10.1111/nph.17421)
Supplement: Supporting Information [file EMS126083-supplement-Supporting_Information.pdf]

Supporting Information

Additional Supporting Information may be found online in the Supporting Information section at the end of the article.

Fig. S1 Layout of the 16 plots along the fertility gradient.

Fig. S2 Principal component analysis (PCA) analysing Ellenberg indicator values of vascular plants of the ground vegetation layer.

Fig. S3 Relationship between fertility index and mineral soil organic C concentration, N concentration, inorganic C concentration, pH, bulk density, stone content, moisture and temperature, respectively.

Fig. S4 Relationship between fertility index and selected fungal guild ratios.

Methods S1 Determination of total mineral soil organic carbon and nitrogen stocks.

Methods S2 Calculation of integrative fertility index based on Ellenberg indicator values.

Table S1 List of vascular plant species in the ground vegetation layer and their Ellenberg indicator values.

Table S2 Total mineral soil organic C and N stocks.

Table S3 List of fungal taxonomic groups including abundance and lifestyle/guild.

© 2021 The Authors
New Phytologist © 2021 New Phytologist Foundation

Table S4 Relationship between relative abundance of selected soil fungal guilds, guild ratios, and microbial respiration, and potential enzyme activities in mineral soil.

Table S5 Relationship between relative abundance of soil fungal guilds and microbial respiration, and potential enzyme activities per g soil C.

Please note: Wiley Blackwell are not responsible for the content or functionality of any Supporting Information supplied by the authors. Any queries (other than missing material) should be directed to the *New Phytologist* Central Office.

About *New Phytologist*

- *New Phytologist* is an electronic (online-only) journal owned by the New Phytologist Foundation, a **not-for-profit organization** dedicated to the promotion of plant science, facilitating projects from symposia to free access for our Tansley reviews and Tansley insights.
- Regular papers, Letters, Viewpoints, Research reviews, Rapid reports and both Modelling/Theory and Methods papers are encouraged. We are committed to rapid processing, from online submission through to publication 'as ready' via *Early View* – our average time to decision is <26 days. There are **no page or colour charges** and a PDF version will be provided for each article.
- The journal is available online at Wiley Online Library. Visit **www.newphytologist.com** to search the articles and register for table of contents email alerts.
- If you have any questions, do get in touch with Central Office (np-centraloffice@lancaster.ac.uk) or, if it is more convenient, our USA Office (np-usaoffice@lancaster.ac.uk)
- For submission instructions, subscription and all the latest information visit **www.newphytologist.com**

© 2021 The Authors
New Phytologist © 2021 New Phytologist Foundation
